# Restenosis After Lacrimal Stent Intubation in a Patient With Indigo Carmine Positivity: A Case Report

**DOI:** 10.7759/cureus.92691

**Published:** 2025-09-19

**Authors:** Kosuke Aonuma, Hiroki Kaneko

**Affiliations:** 1 Department of Ophthalmology, Hamamatsu University School of Medicine, Hamamatsu, JPN

**Keywords:** chronic dacryocystitis, dacryocystorhinostomy, dacryoendoscopy, indigo carmine staining, lacrimal intubation, lacrimal mucosa fibrosis, nasolacrimal duct obstruction, primary acquired nasolacrimal duct obstruction, restenosis, silicone tube

## Abstract

Nasolacrimal duct obstruction (NLDO) is commonly treated with lacrimal stent intubation, yet restenosis remains a frequent challenge. Indigo carmine staining has been reported in research settings as an adjunct in dacryoendoscopy for evaluating mucosal integrity, mainly after tube removal. However, its clinical application has not yet been established, and there are no reports of routine use in daily practice. We present the case of a 55-year-old woman who presented with epiphora, discharge, and itching of the left eye, with a history of lacrimal intubation 10 years earlier. At our clinic, dacryoendoscopy revealed right common canalicular obstruction and left NLDO with clinical features consistent with chronic dacryocystitis, for which bilateral lacrimal intubation was performed. Eight weeks later, the tubes were removed, and dacryoendoscopy was repeated. Indigo carmine (2 mg/0.5 mL) instillation revealed focal staining of the left nasolacrimal mucosa, consistent with localized fibrosis. Six weeks after tube removal, restenosis developed in the left lacrimal drainage system, and the patient was referred to another hospital for dacryocystorhinostomy. This case highlights the potential of indigo carmine staining, performed after lacrimal intubation, in detecting fibrotic mucosa at risk of restenosis. Although promising, its clinical use has not yet been implemented, and further studies are required to establish its prognostic role in NLDO management.

## Introduction

Nasolacrimal duct obstruction (NLDO) is a common condition that often leads to chronic epiphora, recurrent infections, and impaired quality of life. Lacrimal stent intubation is widely accepted as a first-line surgical option for adults with primary acquired nasolacrimal duct obstruction (PANDO), especially in cases without extensive fibrosis, because of its minimally invasive nature and relatively high success rate. However, restenosis after tube removal is not uncommon, particularly in patients with chronic dacryocystitis or advanced mucosal disease. Identifying patients at high risk of recurrence remains a major clinical challenge.

Indigo carmine staining has recently been introduced in research settings as an adjunctive method in dacryoendoscopy to evaluate lacrimal mucosa. Mimura et al. demonstrated that indigo carmine positivity correlates with histopathological findings of advanced fibrosis, including epithelial atrophy, goblet cell depletion, and subepithelial scarring, whereas negative staining was associated with inflammatory stages, suggesting a reversible process with preserved regenerative potential [1]. Despite these insights, indigo carmine has not yet been implemented in routine clinical practice, and, to date, no reports exist describing its widespread clinical use for postoperative evaluation in NLDO patients.

Here, we report a case of NLDO with chronic dacryocystitis in which indigo carmine staining, performed after lacrimal tube removal, revealed localized mucosal fibrosis. The patient subsequently developed restenosis of the left lacrimal drainage system. This case underscores both the potential and current limitations of indigo carmine staining in predicting postoperative outcomes.

## Case presentation

A 55-year-old woman presented to a local clinic with complaints of ocular discharge, itching, and epiphora in the left eye. She had a history of lacrimal silicone tube intubation in the left eye performed at another hospital approximately 10 years earlier. There was no history of preservative-containing topical medications, chronic rhinosinusitis, or systemic inflammatory disease.

At the initial examination in our hospital, lacrimal irrigation with normal saline revealed reflux without purulent discharge in the right eye and reflux with purulent discharge in the left eye. Tear meniscus height was elevated bilaterally.

Three weeks after the initial visit, lacrimal silicone tube intubation was performed under dacryoendoscopic guidance. After local anesthesia around the puncta and lacrimal sac, punctal dilatation was performed with a dilator, and a dacryoendoscope was introduced into the lacrimal drainage system. In the right eye, a common canalicular obstruction was identified and successfully recanalized using direct endoscopic probing. In the left eye, mucoid and blood-stained secretions, suggestive of chronic inflammation, were observed in the lacrimal sac and nasolacrimal duct, with complete NLDO. The obstruction was relieved using a sheath-guided endoscopic probing technique. Silicone tubes were subsequently placed bilaterally under sheath guidance. The final diagnoses were common canalicular obstruction in the right eye and NLDO with clinical features consistent with chronic dacryocystitis in the left eye, although infection was not microbiologically confirmed. Postoperatively, the patient received topical corticosteroids and antibiotics four times daily. Lacrimal irrigation with 3 mL of saline was performed every two weeks to maintain patency.

**Figure 1:**
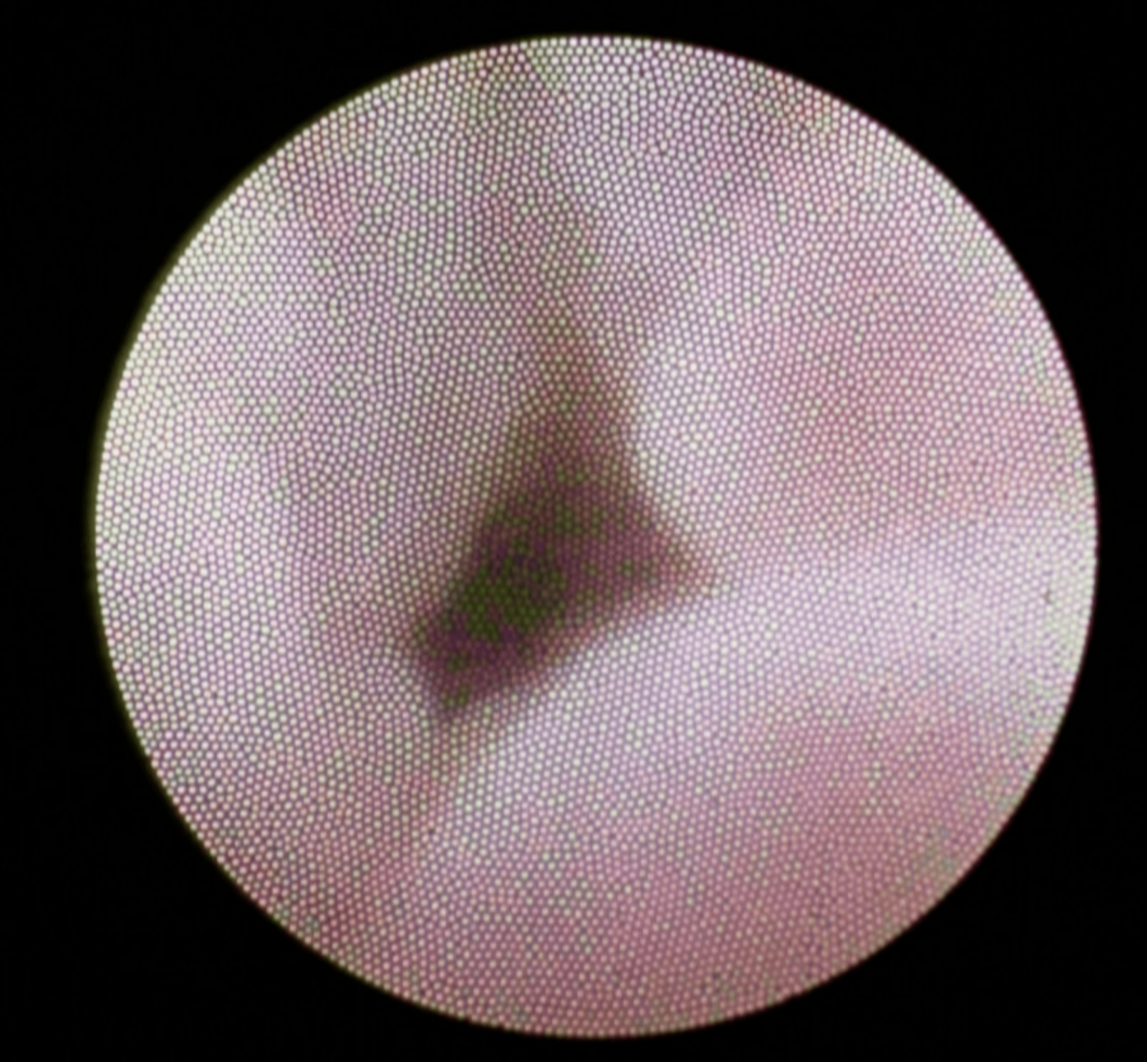
After tube removal, the left nasolacrimal duct remains patent.

**Figure 2:**
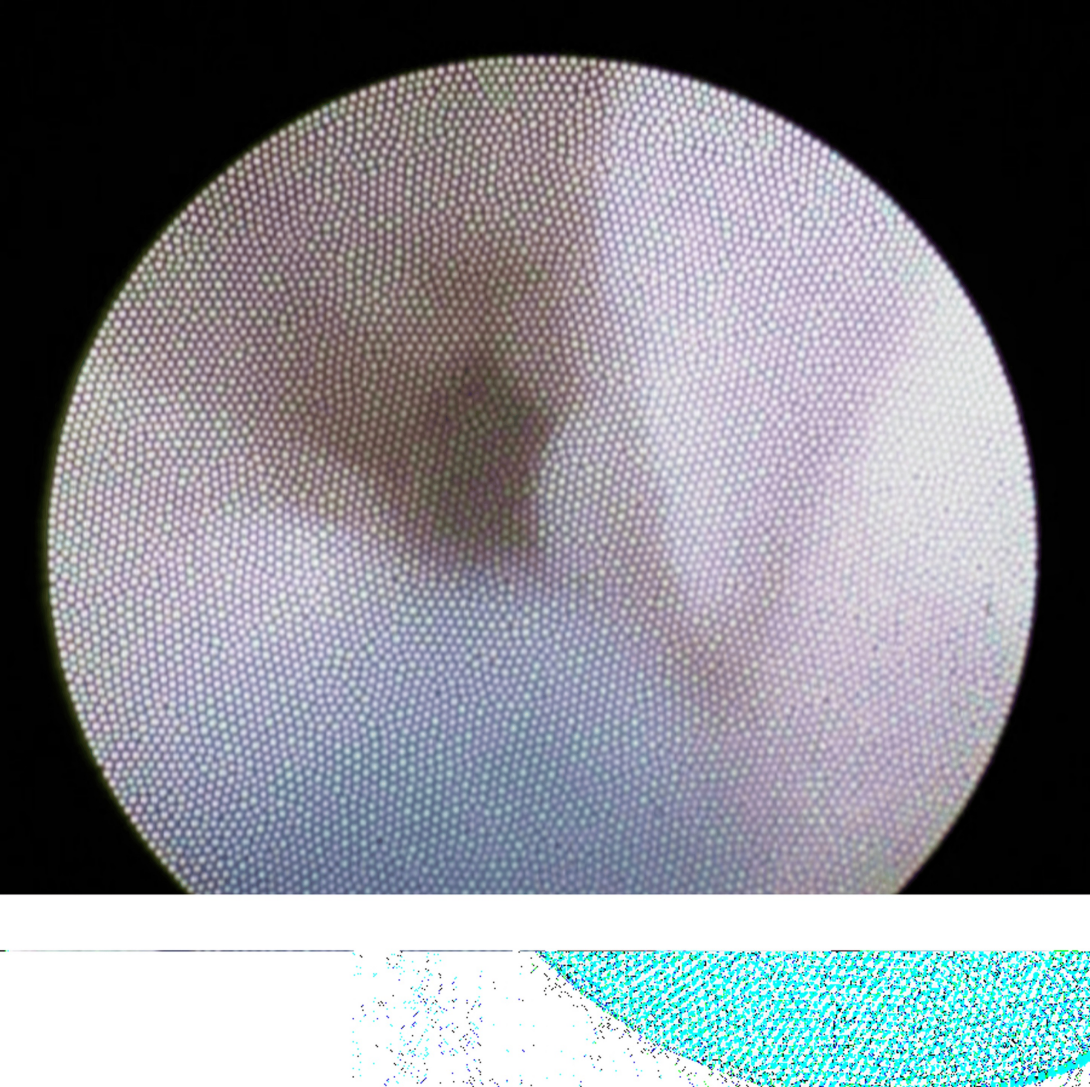
Indigo carmine staining of the left nasolacrimal duct showing focal mucosal staining, indicating localized fibrosis.

Eight weeks after surgery, the tubes were removed, and dacryoendoscopic evaluation was repeated. Following lavage with distilled water, indigo carmine solution (2 mg/0.5 mL) was instilled through the irrigation channel of the dacryoendoscope and immediately washed out with distilled water to remove excess dye. Both lacrimal drainage systems were confirmed to be patent (Figure 1). The right side showed no staining, while the left nasolacrimal duct was mostly unstained except for a focal area of blue staining along the mucosa (Figure 2). This finding was explained to the patient as evidence of localized mucosal fibrosis, suggesting an increased risk of restenosis.

Regular irrigation with saline was continued after tube removal. Although tear meniscus height initially remained stable, six weeks later, the left eye showed increased reflux during irrigation, indicating recurrent NLDO. Further intervention, such as dacryocystorhinostomy, was considered, and the patient was referred to a specialized tertiary facility.

## Discussion

Lacrimal stent intubation is widely used for the treatment of NLDO. Nevertheless, recurrence remains a major challenge, particularly in patients with chronic dacryocystitis. Known risk factors include chronic inflammation, progressive mucosal fibrosis, and long-standing obstruction. Our case illustrates that even after technically successful intubation and initial patency, restenosis may still occur due to subtle mucosal changes.

Clinical studies have clarified the indications for silicone intubation. Mimura et al. reported that Nunchaku-style silicone tube intubation achieved excellent success in upper obstruction (94.6%) and in lower obstruction without dacryocystitis (82.9%), but outcomes were significantly worse when dacryocystitis was present (52.4%). In such cases, endonasal dacryocystorhinostomy achieved much higher success (95.5%) [2]. These findings indicate that while intubation is highly effective in early or uncomplicated PANDO, chronic inflammation and fibrosis substantially reduce long-term success.

Indigo carmine staining has been reported in experimental and research settings as an adjunct in dacryoendoscopy. Mimura et al. demonstrated that indigo carmine positivity correlates strongly with advanced fibrotic remodeling of the lacrimal mucosa, including epithelial atrophy and subepithelial scarring, while negative staining corresponds to earlier inflammatory stages with preserved regenerative potential [1]. Importantly, indigo carmine staining does not indicate infection or the presence of pus; rather, it reflects the degree of mucosal fibrosis and epithelial degeneration. Although promising, indigo carmine staining remains an experimental and exploratory technique, supported only by preliminary research. It has not been adopted into routine clinical practice, and no reports describe its widespread use. Our case, therefore, represents a rare example in which indigo carmine was applied after tube removal, with focal staining suggesting, but not definitively predicting, subsequent restenosis. This finding supports the hypothesis that indigo carmine positivity may identify mucosa at risk for fibrosis-related recurrence. However, as no histopathology or laboratory confirmation was performed in this case, the staining results should be regarded as hypothesis-generating rather than definitive proof of fibrosis. Rather than establishing a new standard of care, this case should be regarded as illustrative of indigo carmine’s potential role and as supportive of earlier research.

Recent evidence further highlights the evolving role of dacryoendoscopy as a platform technology. Wong et al. systematically reviewed 18 studies and confirmed that dacryoendoscopy achieves high treatment success (73.7-100%), with particularly excellent outcomes in congenital NLDO (97-100%) and favorable results in adults (73.7-96.9%). Reported complications were mostly minor, including edema, hemorrhage, and false passage formation, and severe events were rare [3]. In line with this, Kim and Lew found that dacryoendoscopy allowed real-time identification and correction of false passages, which occurred in 2.3% of PANDO cases, thereby improving safety and efficacy [4]. Beyond these clinical advantages, dacryoendoscopy has been increasingly recognized as a frontier technology. Since its introduction in the early 21st century, it has enabled direct visualization of intraluminal pathology and is now standardized, providing a reliable platform for integrating adjunctive techniques such as indigo carmine staining [5].

The duration of silicone tube intubation also plays a critical role in long-term outcomes. Zimmermann et al. demonstrated that removal earlier than three months was significantly associated with higher recurrence (success rate 38% vs. 61% for ≥3 months) [6]. In our case, the tube was removed after two months, and restenosis subsequently developed. This course is consistent with Zimmermann et al.’s findings and suggests that premature removal may compromise mucosal healing and promote fibrosis-related obstruction.

Histopathological investigations add further depth to this interpretation. Makselis et al. analyzed 275 lacrimal sac biopsies and found chronic nongranulomatous inflammation in over 70% of cases, while 1.1% harbored tumors such as adenoid cystic carcinoma, eccrine spiradenoma, and small B-cell lymphoma. Notably, clinical presentation alone did not distinguish inflammatory from neoplastic disease [7]. These results confirm that chronic inflammation and fibrosis dominate NLDO pathology, but rare malignant causes must also be considered. Ali further described PANDO as the outcome of a multifactorial cascade, progressing from recurrent inflammation through epithelial degeneration and goblet cell depletion to subepithelial fibrosis [8]. They also emphasized modifying factors such as anatomical narrowing, vascular changes, hormonal imbalance, microbial influences, and impaired local host defenses, with chronic dacryocystitis accelerating remodeling and worsening outcomes.

Taken together, our case highlights both the promise and the limitations of indigo carmine staining. Positive staining may serve as a prognostic marker for fibrosis-driven recurrence, complementing dacryoendoscopic visualization. Further prospective studies are required to validate its predictive value and establish standardized protocols for clinical use.

A limitation of this report is that dacryoendoscopic images obtained during the procedure were blurred, which is not uncommon in clinical practice. This limited the ability to fully document the intraluminal findings.

## Conclusions

We report a case of NLDO with chronic dacryocystitis in which indigo carmine staining, performed after tube removal, revealed localized fibrosis of the nasolacrimal duct mucosa. The patient subsequently developed restenosis in the left lacrimal drainage system. This case suggests that indigo carmine staining may help identify high-risk mucosa prone to recurrence, although its clinical application remains unestablished. Future prospective studies are necessary to clarify its prognostic value and define its role in the surgical management of NLDO.
